# Correlation of tryptophan metabolites with connectivity of extended central reward network in healthy subjects

**DOI:** 10.1371/journal.pone.0201772

**Published:** 2018-08-06

**Authors:** Vadim Osadchiy, Jennifer S. Labus, Arpana Gupta, Jonathan Jacobs, Cody Ashe-McNalley, Elaine Y. Hsiao, Emeran A. Mayer

**Affiliations:** 1 G. Oppenheimer Center for Neurobiology of Stress and Resilience, UCLA Vatche and Tamar Manoukian Division of Digestive Diseases, Los Angeles, CA, United States of America; 2 UCLA Microbiome Center, David Geffen School of Medicine at UCLA, Los Angeles, CA, United States of America; 3 UCLA Department of Integrative Biology and Physiology, Los Angeles, CA, United States of America; Instituto de Agroquimica y Tecnologia de Alimentos, SPAIN

## Abstract

**Objective:**

A growing body of preclinical and clinical literature suggests that brain-gut-microbiota interactions play an important role in human health and disease, including hedonic food intake and obesity. We performed a tripartite network analysis based on graph theory to test the hypothesis that microbiota-derived fecal metabolites are associated with connectivity of key regions of the brain’s extended reward network and clinical measures related to obesity.

**Methods:**

DTI and resting state fMRI imaging was obtained from 63 healthy subjects with and without elevated body mass index (BMI) (29 males and 34 females). Subjects submitted fecal samples, completed questionnaires to assess anxiety and food addiction, and BMI was recorded.

**Results:**

The study results demonstrate associations between fecal microbiota-derived indole metabolites (indole, indoleacetic acid, and skatole) with measures of functional and anatomical connectivity of the amygdala, nucleus accumbens, and anterior insula, in addition to BMI, food addiction scores (YFAS) and anxiety symptom scores (HAD Anxiety).

**Conclusions:**

The findings support the hypothesis that gut microbiota-derived indole metabolites may influence hedonic food intake and obesity by acting on the extended reward network, specifically the amygdala-nucleus accumbens circuit and the amygdala-anterior insula circuit. These cross sectional, data-driven results provide valuable information for future mechanistic studies.

## Introduction

A growing body of preclinical literature has demonstrated bidirectional signaling between the gut microbiome and the brain, mediated via neural, metabolic, endocrine, and immune-related signaling mechanisms [1, 2]. As the composition of the human gut microbiome is dependent on host diet, there is likely strong selective pressure on the microbiota to modulate food intake and eating habits [3]. In support of this concept are studies on animal models of obesity that demonstrate the gut microbiota’s influence on ingestive behavior [3, 4].

Past studies have shown associations between increased weight and alterations in brain activity and connectivity, underscoring the possible role of the brain in the pathophysiology of obesity [5–9]. Brain networks involving the nucleus accumbens (NAcc), amygdala and the anterior insula (aINS) are among the most extensively studied brain regions with respect to gut-brain signaling involved in the regulation of non-homeostatic food intake [10]. These regions have been shown to play a role in assessing the hedonic value of food [11]. The amygdala integrates affective information with cortical sensory inputs and contains glutamatergic projections to the NAcc [12, 13]. This amygdala-NAcc circuit is important in the promotion of motivational behavioral responses and has been implicated in addiction disorders, and in the regulation of hedonic eating habits (“food addiction”) [12–14]. The aINS plays a role in the experience of emotional feelings and conscious urgings and cravings, in addition to its role as an association cortex, integrating input from subcortical, limbic and executive control brain networks [15, 16].

In addition to identifying anatomical and functional alterations in specific brain regions, more recent efforts have focused on identifying alterations in brain network properties [17]. Brain connectivity can be assessed using complex network analysis via graph theory. Within this framework, brain regions are characterized by measures that quantify their contribution to the functional and anatomical integrity and information flow in the whole brain network [18–21].

Although the exact signaling mechanisms underlying the communication within the brain-gut-microbiome (BGM) axis remain incompletely understood, tryptophan (TRP) metabolites have been implicated as important signaling molecules in this system [22]. Perhaps the most extensively studied TRP metabolite is serotonin (5-HT), with diverse roles in both the gastrointestinal (GI) tract (i.e. secretion and absorption, intestinal transit) and the central nervous system (CNS) (i.e. mood, pain modulation, behavior, cognitive function) [23]. The close proximity of the gut microbiota to 5-HT-producing enterochromaffin cells (ECCs) strongly suggests that the gut microbiota may modulate the serotonergic system. In turn, the intimate, synapse-like contact of vagal afferent nerve terminals to the basolateral side of ECCs establishes a pathway by which microbial signals can activate vagal afferents via 5-HT release. In a mouse model, metabolites derived from spore-forming bacteria of the human gut microbiota have been shown to play a prominent role in the regulation of ECC 5-HT synthesis and release [24]. TRP also acts as a precursor to the kynurenine (KYN) family of molecules. Quantitatively, the KYN pathway is the most important pathway for TRP metabolism, outside of protein synthesis, as under normal conditions it is responsible for more than 90% of TRP catabolism [25].

Though the KYN pathway represents the major TRP degradation pathway by *host* cells, most undigested dietary TRP in the gut lumen is converted by *gut microbes* to indole. This is mediated by the exclusively microbial tryptophanase enzyme, which catalyzes the conversion of TRP to indole, pyruvate, and ammonia [25, 26]. Indole is a common component of human feces, often detectable at concentrations up to 1,100 μM [27]. Indole and indole-derived metabolites have many functions within the BGM axis, including the modulation of KYN synthesis and incretin secretion, strengthening of the mucosal intestinal barrier, and attenuation of CNS inflammation–all of which have been shown to be disrupted in obesity [25, 27–32].

By studying anatomical and functional connectivity measures and fecal gut microbial metabolites in healthy subjects with and without elevated body mass index (BMI), we aimed to test the hypothesis that indole metabolites are associated with connectivity of key regions of the brain’s reward network. We determined the relationship between gut-derived indole metabolites with local connectivity of regions of the extended reward network (amygdala, aINS and NAcc) and clinical measures related to hedonic food intake and body weight. Even though these results do not prove causation, and need to be validated in a larger sample, they provide support for an association between gut-derived microbial metabolites and aspects of brain structure and function, which may play a role in hedonic food intake and obesity. These cross sectional data-driven results provide valuable information for future mechanistic studies.

## Materials and methods

### Subjects

Stool samples were collected at the G. Oppenheimer Center for Neurobiology of Stress and Resilience at the University of California, Los Angeles (UCLA) from 63 right-handed healthy subjects (29 males, 34 females), who also underwent multimodal brain imaging studies. Exclusionary criteria included (1) serious medical conditions or taking medications which could compromise interpretation of the brain imaging; (2) current major psychiatric diagnoses or use of psychotropic medications in the past 6 months; (3) use of antibiotics in the past 3 months and (4) excessive physical exercise (e.g., marathon runners).

Written informed consent was obtained from all subjects, and all subjects were compensated for participating in the study. The study was approved by the UCLA Institutional Review Board and was conducted in full accordance with the institutional guidelines regulating human subjects research.

### Questionnaires

The Hospital Anxiety and Depression (HAD) Scale [33] was obtained to assess anxiety and depression symptoms. Scores 0–7 define the normal range, 8–10 define the borderline range, and 11–21 define the abnormal range [33]. As only two subjects received scores outside of the normal range for the depression subscale, only the anxiety subscale was included in our analysis (see results). All subjects completed the HAD scale. Subjects filled out the Yale Food Addiction Scale (YFAS) questionnaire, a 25-item scale developed to measure food addiction. This scale is based upon the substance dependence criteria found in the DSM-5 (e.g., tolerance [marked increase in amount; marked decrease in effect], withdrawal [agitation, anxiety, physical symptoms], loss of control [eating to the point of feeling physical ill]) [34]. The YFAS has displayed a good internal reliability (Kuder–Richardson *α* = .86) [34]. Only 42 subjects completed the YFAS, as it was not available at the time of recruitment of all subjects. BMI was recorded for all subjects.

### Fecal metabolomics

Fecal samples were stored at -80°C and shipped to Metabolon for processing and analysis as a single batch on their global metabolomics and bioinformatics platform [35]. Data was curated by mass spectrometry using specialized software as previously described [35]. Metabolites of interest were restricted to indole, 3-methylindole (skatole), and indoleacetic acid (IAA).

### Magnetic resonance imaging

#### Structural MRI

High resolution T1-weighted images were acquired on a Siemens Allegra 3 Tesla scanner, repetition time = 2200 ms, echo time = 2.85s, inversion time = 750 ms, flip angle = 20 degrees, Field of view = 220 × 220 mm, resolution = 256 × 256, slices per volume = 176, slice thickness = 1 mm, voxel size = 0.86 × 0.86 × 1 mm.

#### Functional MRI

Resting-state scans between 8m6s and 10m6s in length were acquired with an echo planar sequence with the following parameters: echo time = 28 ms, repetition time = 2000 ms, scan duration = 8m6s–10 m6s, flip angle = 77°, field of view = 220 mm, slices = 40 and slice thickness = 4.0 mm, and slices were obtained with whole-brain coverage.

#### Diffusion-weighted MRI

Diffusion-weighted magnetic resonance imaging was acquired according to two comparable acquisition protocols, in either 61 or 64 noncolinear directions with *b* = 1000 s mm^−2^, with 8 or 1 b = 0 s mm^−2^ images respectively, TR = 9500 ms, TE = 88ms and field of view = 256 mm with an acquisition matrix of 128x128, and a slice thickness of 2 mm to produce 2 × 2 × 2 mm^3^ isotropic voxels.

### Quality control of MRI data

Preprocessing for quality control involved bias field correction, coregistration, motion correction, spatial normalization, tissue segmentation, Fourier transformation for frequency distribution, and specific quantitative checks for DTI images (apparent diffusion coefficient and fractional anisotropy [FA]). Structural images were included based on compliance with acquisition protocol, full brain coverage, minimal motion (Gibbs ringing), absence of flow/zipper, and minor atrophy/vascular degeneration. Functional images were included based on compliance with acquisition protocol, full brain coverage, motion estimate of < ½ voxel size between adjacent time points, low standard deviation across time series for all voxels, ghosting in cerebrum, minimal physiological noise (>0.2Hz in frequency spectrum), and few to no outlier voxels, mean intensity shifts, or K-space “spikes.” Preprocessing for diffusion-weighted imaging included visually checking for artifacts and motion on the raw diffusion weighted and b0 images, visual assessment of FA and mean diffusitivity (MD) map quality, as well checking for physiologically feasible FA and MD values (FA of 0–0.1 and MD of 3–4 μm2/ms in ventricles, and FA of 0.6–0.9 and MD of 0.6–0.9 μm2/ms in splenium of corpus callosum). Maximum relative motion thresholds for translation and rotation for each direction (x, y, and z) were set at 2mm and 2°, respectively. No subjects presented with serious adverse imaging artifacts and no subjects exceeded motion thresholds: the highest maximum relative translation for any subject was 1.37mm, and the highest maximum relative rotation was 1.59°. Only one DTI scan did not meet quality control criteria and was excluded from analysis. All other DTI and resting state scans met quality control criteria and were included in the analysis.

### Structural image parcellation

T1-image segmentation and regional parcellation were conducted using FreeSurfer v.5.3.0[36–38] following the nomenclature described in the Destrieux and Harvard-Oxford subcortical atlas [39, 40]. For each cerebral hemisphere, a set of 74 cortical structures were labeled in addition to 7 subcortical structures and to the cerebellum. One additional midline structure (the brain stem) was also included, for a complete set of 165 parcellations for the entire brain.

### Functional brain network construction

The parcellation and the functional connectivity results were combined to produce a 165x165 weighted, unidirected connectivity matrix. Resting-state image preprocessing was performed using the SPM8 software (Wellcome Department of Cognitive Neurology, London, UK). Images were transformed from DICOM into NIfTI, slice-time corrected, co-registered with the high-resolution structural images, spatially normalized to the MNI space, and resampled to a voxel size of 2 X 2 X 2 mm. Normalized functional images were further preprocessed and analyzed using the SPM-based CONN toolbox version 13 (www.nitrc.org/projects/conn). The resting-state images were filtered using a band-pass filter (0.008/s<f<0.08/s) to reduce the low- and high-frequency noises. A component based noise-correction method, CompCor,[41] was applied to remove nuisances for better sensitivity and specificity of the analysis. Six motion realignment parameters and confounds for white matter and CSF were removed using regression. The connectivity between the 165 brain regions was indexed by a matrix of Fisher z transform correlation coefficients reflecting the association between average temporal BOLD time series signals across all voxels in each brain region. Functional connections were retained at z>.3 and all other values were set to 0. The magnitude of the z-score represents the weights in the functional network.

### Anatomical network construction

Regional parcellation and tractography results were combined to produce a weighted, unidirected connectivity matrix. White matter connectivity for each subject was estimated between the 165 brain regions using DTI fiber tractography,[42] performed via the Fiber Assignment by Continuous Tracking (FACT) algorithm[43] using TrackVis (http://trackvis.org). The final estimate of white matter connectivity between each of the brain regions was determined based on the number of fiber tracts intersecting each region. Weights of the connections were then expressed as the absolute fiber count divided by the individual volumes of the two interconnected regions [20].

### Regions of interest

**Fig 1:**
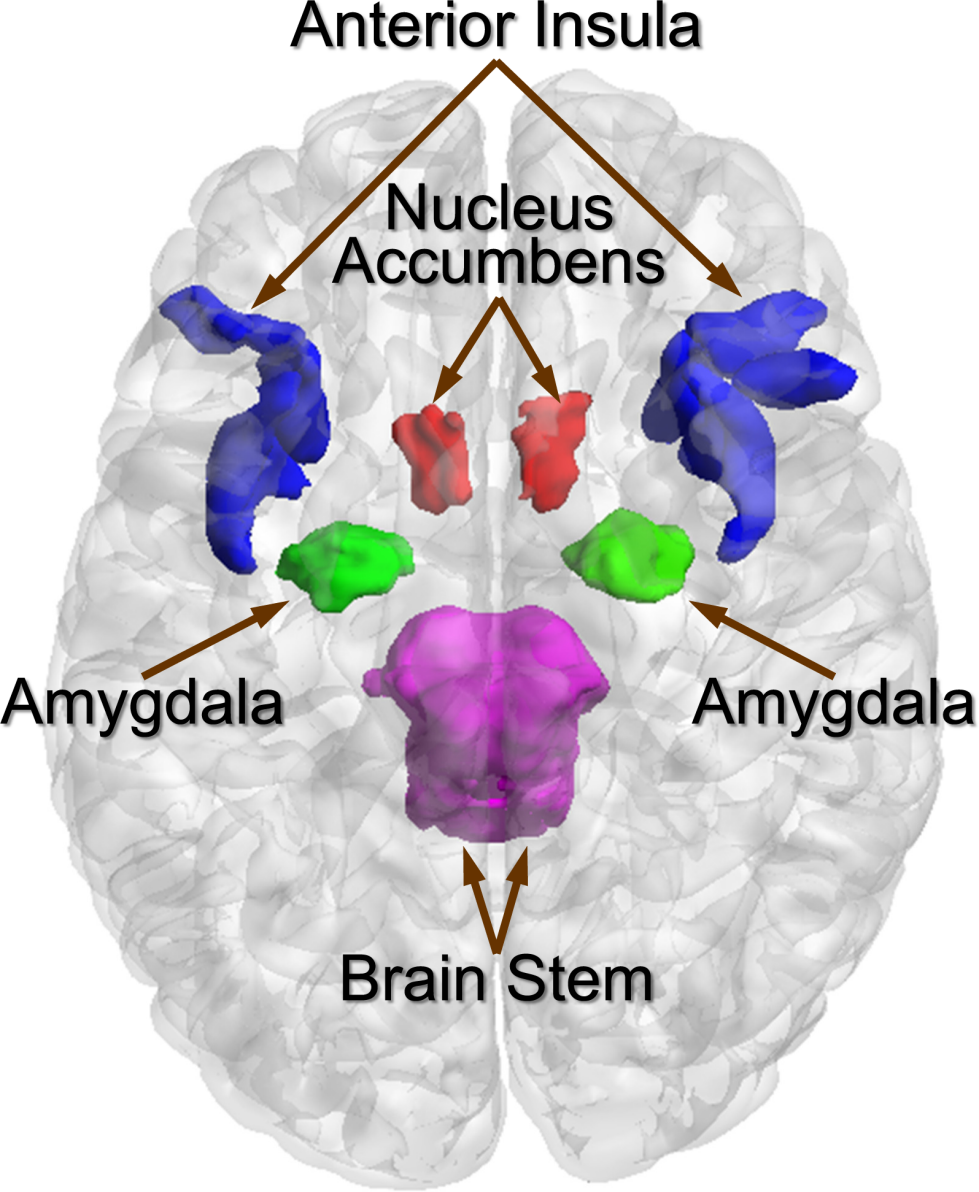
Regions of interest and associated regions. Fig 1 displays regions of interest (nucleus accumbens [orange], amygdala [green], and anterior insula [blue; includes short insular gyrus, anterior segment of the circular sulcus of the insula, horizontal ramus of the anterior segment of the lateral sulcus, and vertical ramus of the anterior segment of the lateral sulcus]) and the brain stem (pink).

Key regions of the extended reward that have been extensively studied with respect to gut-brain signaling involved in the regulation of non-homeostatic food intake were included [10]. Regions of interest were restricted to the NAcc, amygdala, and aINS (including short insular gyrus, anterior segment of the circular sulcus of the insula, horizontal ramus of the anterior segment of the lateral sulcus, and vertical ramus of the anterior segment of the lateral sulcus) (**Fig 1**).

### Computing network metrics

The Graph Theoretic GLM tool (www.nitrc.org/projects/metalab_gtg)[44] and in-house matlab scripts were used to calculate and analyze the brain network properties and organization from the subject-specific functional brain networks for the brain regions of interest.

Regions with high centrality are highly influential, communicate with many other regions, facilitate functional integration, and play a key role in network resilience to insult [21]. Two indices of centrality were computed: 1) *Degree Strength* reflects the number of other regions a brain region interacts with functionally (local prominence) and *2) Betweenness centrality*, reflecting the ability of a region to influence information flow (signaling) between two other regions.

### Statistical analysis

Tripartite network analysis was performed to integrate information from three data sets: 1) Stool derived indole metabolites (indole, skatole, and IAA), 2) clinical data (BMI, YFAS, HAD Anxiety) and 3) the functional and anatomical network metrics characterizing regions of interest (amygdala, NAcc, and aINS [short insular gyrus, anterior segment of the circular sulcus of the insula, horizontal ramus of the anterior segment of the lateral sulcus, and vertical ramus of the anterior segment of the lateral sulcus]). The interaction between the phenome (clinical measures), microbiome (stool-derived indole metabolites) and connectome (brain connectivity) was determined by computing Spearman correlations between different data types controlling for age and sex in Matlab version R2015b. The unweighted force-directed layout in Cytoscape v. 3.5.1 was used to visualize and construct brain, symptom, and gut-derived metabolite interaction networks thresholded at *q* < .05 corrected *p* value using a false discovery rate of 5%. We present the networks based on selecting the metabolites and their first neighbors as nodes and all adjacent edges. The results are described in terms of direct (correlations with metabolites) and indirect effects (clinical symptoms and functional and anatomical network metrics that are a part of the interaction network but not directly correlated with the metabolite).

## Results

### Clinical and behavioral characteristics

Patient ages ranged from 18 to 60 (mean: 29.42, SD: 10.76). BMI ranged from 17.90 to 37.34 (mean: 25.82, SD: 4.93). Subjects with high BMI (BMI ≥ 25: mean = 30.00, SD = 3.31, range = 25.11–37.34) consisted of 17 males (mean = 29.26, SD = 3.33, range = 25.11–37.34) and 14 females (mean = 30.90, SD = 3.18, range = 25.52–37.09). Subjects with normal BMI (BMI < 25: mean BMI = 21.77, SD = 1.90, range = 17.90–24.53) consisted of 12 males (mean = 22.21, SD = 1.88, range = 17.90–24.10) and 20 females (mean = 21.50, SD = 1.91, range = 18.80–24.53). Scores on the YFAS ranged from 0 to 7 (mean: 1.24, SD: 1.25). HAD Anxiety scores ranged from 0 to 13 (mean: 4.25, SD: 3.41; normal: 50 subjects, borderline: 10 subjects, abnormal: 3 subjects). HAD Depression scores ranged from 0 to 11 (mean: 1.87, SD: 2.23; normal: 61 subjects, borderline: 1 subject, abnormal: 1 subject).

### Association of clinical parameters with indole metabolites

The indole metabolite skatole positively correlated with YFAS scores. Although not surviving FDR correction, indole positively correlated with BMI and negatively with anxiety scores. (For details, see **Tables 1 and 2**)

**Table 1:** Amygdala-nucleus accumbens associations.

**Metabolite**	**Functional Connectivity**	***r***	***p***	***q***	***df***
Indole	B_R_NAcc	0.59608	< 0.001	**< 0.001**	62
IAA	S_R_Amg	0.37640	0.003	**0.011**	62
Indole	S_R_NAcc	0.36637	0.004	**0.011**	62
IAA	S_R_NAcc	0.33201	0.009	**0.018**	62
Skatole	S_R_NAcc	0.31022	0.015	**0.022**	62
Skatole	S_L_NAcc	0.33839	0.008	**0.046**	62
**Clinical/Behavioral Measure**	**Functional Connectivity**	***r***	***p***	***q***	***df***
YFAS	S_L_NAcc	0.59632	< 0.001	**< 0.001**	41
ANX	S_L_NAcc	0.29999	0.019	0.056	62
YFAS	S_R_NAcc	0.36944	0.019	0.114	62
**Metabolite**	**Anatomical Connectivity**	***r***	***p***	***q***	***df***
Skatole	S_L_Amg	0.55362	< 0.001	**< 0.001**	61
**Metabolite**	**Clinical/Behavioral Measure**	***r***	***p***	***q***	***df***
Skatole	YFAS	0.48043	0.002	**0.017**	41
Indole	ANX	-0.28741	0.025	0.086	62
Indole	BMI	0.28036	0.029	0.090	62

This table shows all significant associations prior to FDR correction with r, p, FDR-corrected q values, and df (degrees of freedom) for the amygdala-nucleus accumbens circuit.

Abbreviations: ANX, Hospital anxiety and depression (HAD) scale anxiety score; B_R_NAcc, Betweenness centrality of the right nucleus accumbens; BMI, Body mass index; IAA, Indoleacetic acid; S_L_Amg, Degree strength of the left amygdala; S_L_NAcc, Degree strength of the left nucleus accumbens; S_R_Amg, Degree strength of the right amygdala; S_R_NAcc, Degree strength of the right nucleus accumbens; YFAS, Yale food addiction scale score.

**Table 2:** Amygdala-anterior insula associations.

**Metabolite**	**Functional Connectivity**	***r***	***p***	***q***	***df***
IAA	S_R_Amg	0.37639	0.003	**0.042**	62
Skatole	B_L_ALSVerp (aINS)	0.33542	0.008	0.123	62
IAA	S_R_ALSVerp (aINS)	0.27091	0.035	0.206	62
IAA	S_L_ALSHorp (aINS)	0.26168	0.042	0.625	62
**Clinical/Behavioral Measure**	**Functional Connectivity**	***r***	***p***	***q***	***df***
YFAS	B_L_ALSVerp (aINS)	0.53843	< 0.001	**0.005**	41
BMI	B_R_ShoInG (aINS)	0.29197	0.022	0.336	62
**Metabolite**	**Anatomical Connectivity**	***r***	***p***	***q***	***df***
Skatole	S_L_Amg	0.55362	< 0.001	**< 0.001**	61
Indole	B_L_ALSHorp (aINS)	0.49712	< 0.001	**< 0.001**	61
Skatole	S_R_ALSHorp (aINS)	0.31036	0.016	0.237	61
Skatole	S_R_ALSVerp (aINS)	0.26351	0.042	0.314	61
Skatole	B_R_ALSHorp (aINS)	0.25465	< 0.050	0.503	61
**Clinical/Behavioral Measure**	**Anatomical Connectivity**	***r***	***p***	***q***	***df***
BMI	B_L_ShoInG (aINS)	0.30157	0.019	0.288	61
**Metabolite**	**Clinical/Behavioral Measure**	***r***	***p***	***q***	***df***
Skatole	YFAS	0.48043	0.002	**0.017**	41
Indole	ANX	-0.28741	0.025	0.086	62
Indole	BMI	0.28036	0.029	0.090	62

This table shows all significant associations prior to FDR correction with r, p, FDR-corrected q values, and df (degrees of freedom) for the amygdala-anterior insula circuit.

Abbreviations: ANX, Hospital anxiety and depression (HAD) scale anxiety score; B_L_ALSHorp, Betweenness centrality of the left horizontal ramus of the anterior segment of the lateral sulcus (aINS); B_L_ALSVerp, Betweenness centrality of the left vertical ramus of the anterior segment of the lateral sulcus (aINS); B_L_ShoInG, Betweenness centrality of the left short insular gyrus (aINS); B_R_ALSHorp, Betweenness centrality of the right horizontal ramus of the anterior segment of the lateral sulcus (aINS); B_R_ShoInG, Betweenness centrality of the right short insular gyrus (aINS); BMI, Body mass index; IAA, Indoleacetic acid; S_L_ALSHorp, Degree strength of the left horizontal ramus of the anterior segment of the lateral sulcus (aINS); S_L_Amg, Degree strength of the left amygdala; S_R_ALSHorp, Degree strength of the right horizontal ramus of the anterior segment of the lateral sulcus (aINS); S_R_ALSVerp, Degree strength of the right vertical ramus of the anterior segment of the lateral sulcus (aINS); S_R_Amg, Degree strength of the right amygdala; YFAS, Yale food addiction scale score.

### Amygdala-NAcc circuit

Indole, skatole and IAA showed large positive correlations with *functional* connectivity of the NAcc. Skatole showed a positive association with measures of *anatomical* connectivity of the amygdala, whereas IAA associated positively with *functional* connectivity of the amygdala. YFAS scores and HAD Anxiety scores showed positive associations with *functional* connectivity of the NAcc. **(see Table 1)**

### Amygdala-aINS circuit

*Anatomical* connectivity of the amygdala showed a positive correlation with skatole, while *functional* connectivity of this brain regions showed a positive association with IAA. Anatomical connectivity of a region within the aINS, showed a positive association with indole, while functional connectivity of the same brain region showed a positive association with YFAS scores. (see **Table 2**)

**Fig 2:**
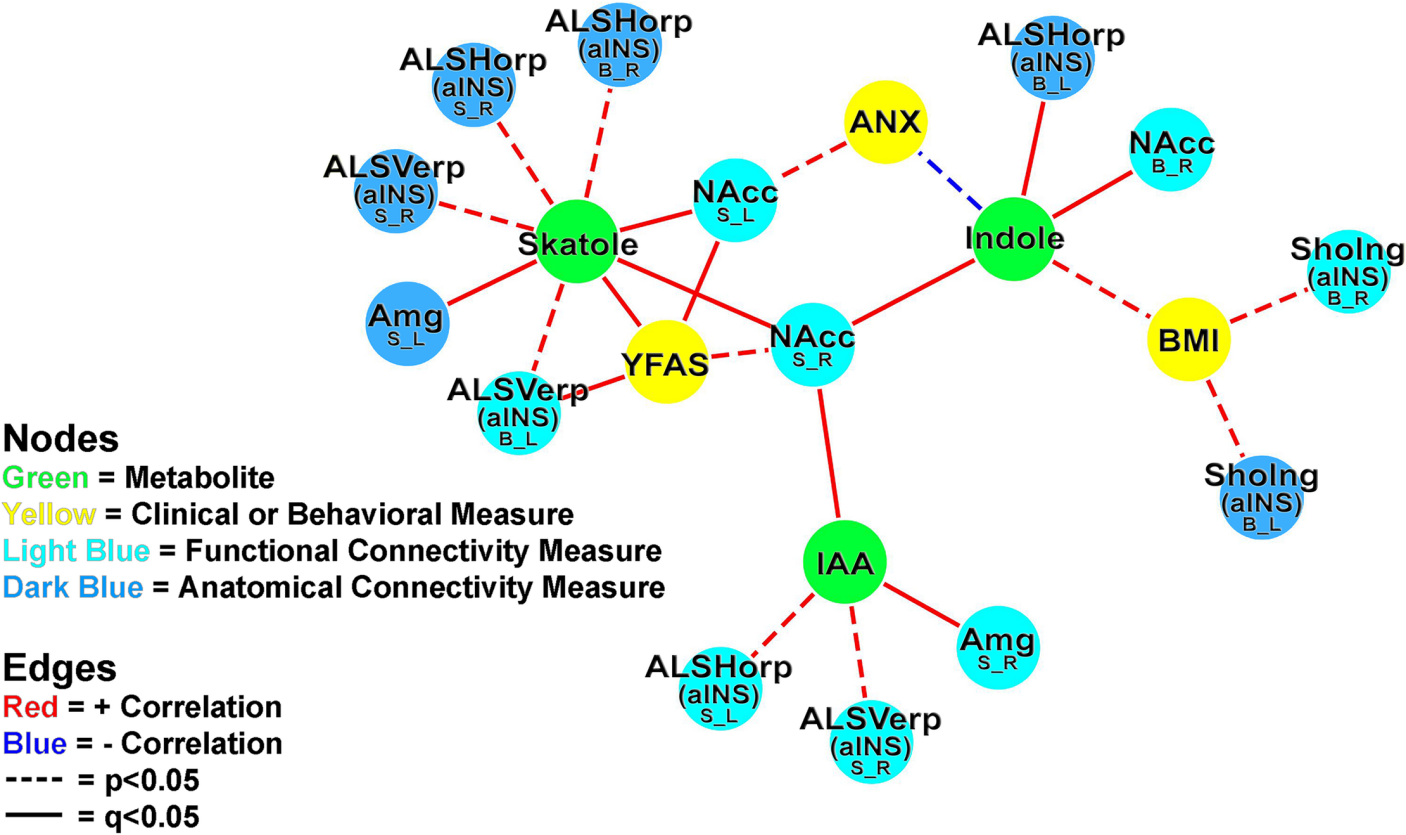
Tripartite association network. Fig 2 displays the tripartite association network between indole metabolites, clinical and behavioral measures, and functional and anatomical connectivity of the amygdala-NAcc circuit and the amygdala-aINS circuit. All significant (p < .05) associations are included in this visualization. Functional brain connectivity of regions of interest is presented with the region of interest noted in a larger font, with the connectivity measure and lateralization indicated below in the form X_Y, where X indicates a connectivity measure (B, Betweenness centrality; S, Degree strength) and Y indicates lateralization (L, Left; R, Right). Abbreviations: aINS, anterior insula; ALSHorp, horizontal ramus of the anterior segment of the lateral sulcus (aINS); ALSVerp, vertical ramus of the anterior segment of the lateral sulcus (aINS); Amg, amygdala; ANX, Hospital anxiety and depression (HAD) scale anxiety score; BMI, Body mass index; IAA, Indoleacetic acid; NAcc, nucleus accumbens; ShoInG, short insular gyrus (aINS); YFAS, Yale food addiction scale score.

**Fig 2** displays the tripartite association network between indole metabolites, clinical and behavioral measures, and connectivity of the amygdala-aINS circuit and the amygdala-NAcc circuit. All three metabolites showed indirect association with YFAS through functional connectivity of the right NAcc. Skatole also showed an indirect association with anxiety through functional connectivity of the left NAcc, in addition to indirect associations to YFAS through functional connectivity of the left NAcc and a region within the aINS.

## Discussion

This study demonstrates associations between fecal indole metabolites, measures of functional and anatomical connectivity of key regions of the extended reward network, in addition to BMI, food addiction scores (YFAS) and anxiety symptom scores (HAD Anxiety) in healthy individuals with varying BMIs. These results are consistent with the literature on the role of indole metabolites in mammalian physiology and support the hypothesis that gut microbiota-derived indole metabolites may influence food intake by acting on the extended reward network, specifically the amygdala-NAcc circuit and the amygdala-aINS circuit. Even though these cross sectional findings were obtained in a relatively small sample cohort, to our knowledge, this is the first report demonstrating an association between microbial-derived stool metabolites of the indole family with connectivity of brain regions of the extended reward network in healthy humans.

### Association of indole metabolites with connectivity of amygdala and nucleus accumbens

Our results show direct associations between the functional connectivity of the amygdala with IAA, and between anatomical connectivity of this region with skatole, with all metabolites showing a strong association with functional connectivity of the NAcc. Skatole showed a direct positive association with YFAS scores, while IAA showed an indirect positive association with skatole through functional connectivity of the NAcc. Although not statistically significant following FDR correction, indole showed a direct positive association with BMI and an indirect positive association with YFAS through functional connectivity of the NAcc. One likely mechanism by which indoles could influence this part of the reward circuit may be through interactions with the enteroendocrine L-cells of the gut and GLP-1. The peptide hormone GLP-1 and GLP-1 receptor agonists have been studied within the context of obesity, as they are associated with better glycemic control, reduction of body weight and increased satiety [45]. Indole can modulate GLP-1 secretion in mouse colonic L-cells by influencing voltage-gated potassium channels and mitochondrial NADH dehydrogenase [29]. Exposure to indole over short periods of time resulted in increased secretion of GLP-1, whereas longer exposure led to decreased secretion [29]. As most GLP-1 is rapidly inactivated by dipeptidyl peptidase 4 prior to leaving the gut, it is likely that GLP-1 acts locally on vagal afferent nerve terminals, and signals via vagal afferents to the nucleus tractus solitarius (NTS) and to brain circuits involved in the regulation of ingestive behavior [45]. This is consistent with a recent human study, which showed that the GLP-1 agonist Exenatide can impact appetite control by modulating the functional connectivity of reward regions associated with the NTS [46]. An alternative hypothesis posits that vagal afferent modulation of the amygdala-NAcc circuit may occur through modulation of the gut serotonergic system. Since it has been shown that metabolites from spore-forming bacteria of the gut microbiota regulate ECC 5-HT synthesis and release, an indole derivative that affects this microbial community may influence the gut serotonergic system [24]. The established role of indoles as valuable intercellular signaling molecules in microbial communities, with examples of indole signaling among spore-forming bacteria, further supports this mechanism [47]. As 5-HT is intimately involved in vagal signaling along the gut-brain axis, it is likely that vagal afferents would be responsible for communicating indole-mediated modulation of the gut serotonergic system to central reward regions [48].

### Association of indole metabolites with connectivity of amygdala and aINS

We found an association between the anatomical connectivity of the aINS with indole levels in the stool, and of the amygdala with fecal skatole levels. In addition, the functional connectivity of the amygdala was associated with fecal IAA levels. Signaling of gut microbiota to the amygdala-aINS circuit may act directly through the amygdala by modulating CNS 5-HT synthesis; one mechanism may involve the aryl hydrocarbon receptor (AhR). Many studies on the effects of indoles on mammalian physiology have focused on their role as AhR ligands. When activated, AhR can function as a transcription factor that regulates the rate limiting enzymes in TRP metabolism along the KYN pathway [49]. Increases in systemic KYN levels can limit peripheral TRP bioavailablity for CNS 5-HT synthesis; KYN competes with TRP to cross the blood-brain barrier (BBB) through the Large Neutral Amino Acid Transporter LAT1, an important point considering TRP uptake is the rate-limiting step in CNS 5-HT synthesis [50]. The consequences of reduced CNS 5-HT have been interrogated through acute TRP depletion (ATD) studies, which generally show that ATD negatively influences mood and feeding behavior as well as visceral sensitivity and abdominal symptoms [51, 52]. Considering the extensive serotonergic innervation of the amygdala and its connections to the INS, modulation of CNS TRP metabolism via indole-activated AhR may explain the association between indoles and the amygdala-aINS circuit and the positive association between skatole and food addiction scores [53].

Indoles may also act on the amygdala-aINS circuit by modulating aINS-mediated gustatory processing. One mechanism that allows the GI tract to constantly sample the gut lumen is through taste receptors expressed on ECCs and L-cells [54, 55]. Considering the established relationship between indole and L-cells, and the proposed relationship between indoles and ECCs, the gut microbiota may represent an extension of this luminal gustatory sensing mechanism. As an example, glucose has been shown to inhibit indole biosynthesis via catabolite repression of the tryptophanase enzyme, whereas TRP has been shown to induce the tryptophanase enzyme [26, 56]. The responsiveness of tryptophanase to the luminal environment underscores the dynamic nature of this indole-aINS gustatory association suggested by our data.

### Association of indole metabolites with clinical and behavioral measures

Fecal skatole levels showed a positive association with food addiction scores; although not significant following FDR correction, fecal indole levels positively associated with BMI and negatively with anxiety scores. In contrast, IAA showed no direct associations with any clinical and behavioral measures. It is interesting to note that IAA is the only metabolite investigated that acts as a true AhR agonist, while both indole and skatole act as weak AhR agonists or partial antagonists [57, 58]. Considering the diversity of AhR ligands that extends even past the indole family of molecules, elevated concentrations of indole and skatole would interfere with the binding of potentially higher affinity ligands to AhR [59]. The balance of indole and skatole to IAA or to other AhR agonists may play an important role in the activity of AhR and subsequent downstream consequences [57], [60], [61]. Although future work is necessary to examine the importance of AhR on the gut-brain axis, our results suggest that indoles with partial-antagonistic properties may be more clinically relevant than indoles with pure agonist properties with respect to the AhR.

### Limitations

As we were interested in gut-microbiota derived indole metabolites, our study only examined fecal samples and did not address possible direct actions of indole on the CNS through circumventricular organs or by crossing the blood brain barrier. Because indoles are prone to extensive modification through the CYP450 system, fecal samples are likely a more accurate representation of the GI metabolomic profile [62]. Although there was a fair distribution and range of BMIs among study participants, our study consisted primarily of subjects with BMIs greater than 18.5. Additionally, less than a third of subjects did not complete the YFAS survey, as it was not available at the time of their recruitment. Due to the limited sample size, we did not explore sex-differences in this study. Future studies with a larger sample size, and perhaps a more heterogeneous subject population with respect to BMI and YFAS scores would need to be conducted to validate our results. Future studies should focus on identifying associations of metabolites with brainstem regions, in particular the NTS in the analysis, possibly by using tailored 3T brainstem acquisition protocols and ultra high field MRI (T7 or higher) [63–65]. Although the associations presented in this study are consistent with conclusions from studies that utilized animal models or *in vitro* approaches, no causative relationships can be implied from an association study.

### Summary and conclusions

To our knowledge, this is the first study demonstrating an association between microbial-derived stool metabolites with connectivity of brain regions of the extended reward network in healthy human subjects with varying BMI and food cravings. It is likely that a combination of some or all of the proposed mechanisms plays a role in these associations. The inducible and repressible nature of the tryptophanase enzyme, combined with the varying binding affinities of different indoles to AhR underscore the importance of metabolic homeostasis of microbiota-derived metabolites and provides further evidence for the dynamic nature of the BGM axis. This exploratory study provides valuable information for future mechanistic studies.

## Supporting information

S1 File DTI data. (XLSX)

S2 File RS data. (XLSX)

## Abbreviations

5-HT: serotonin
AhR: aryl hydrocarbon receptor
aINS: anterior insula
ATD: acute tryptophan depletion
BBB: blood-brain barrier
BGM: brain-gut-microbiome
BMI: body mass index
CNS: central nervous system
ECC: enterochromaffin cell
FA: fractional anisotropy
FACT: fiber assignment by continuous tracking
GI: gastrointestinal tract
HAD: Hospital Anxiety and Depression
IAA: indoleacetic acid
KYN: kynurenine
MD: mean diffusivity
NAcc: nucleus accumbens
NTS: nucleus tractus solitaries
TRP: tryptophan
YFAS: Yale Food Addiction Scale
